# Monitoring and management of chronic kidney disease in ambulatory care – analysis of clinical and claims data from a population-based study

**DOI:** 10.1186/s12913-022-08691-y

**Published:** 2022-11-09

**Authors:** Gesine Weckmann, Janine Wirkner, Elisa Kasbohm, Carolin Zimak, Annekathrin Haase, Jean-François Chenot, Carsten Oliver Schmidt, Sylvia Stracke

**Affiliations:** 1 Department of General Practice, Institute for Community Medicine, University Medicine Greifswald, Fleischmannstrasse 6, 17475 Greifswald, Germany; 2 Faculty of Applied Health Sciences, European University of Applied Sciences, Rostock, Germany; 3 Department of Clinical Psychology and Psychotherapy, Institute for Psychology, University of Greifswald, Greifswald, Germany; 4 Department SHIP - Clinical Epidemiological Research, Institute for Community Medicine, University Medicine, Greifswald, Germany; 5 University Medicine Greifswald, Internal Medicine A, Nephrology, Germany; 6 KfH Center for Dialysis and Transplantation Greifswald, Greifswald, Germany

**Keywords:** Chronic kidney disease, Management, Primary care, Nephrology, Ambulatory care

## Abstract

**Background:**

Although chronic kidney disease (CKD) is highly prevalent in the general population, little research has been conducted on CKD management in ambulatory care.

Objective was to assess management and quality of care by evaluating CKD coding in ambulatory care, patient diagnosis awareness, frequency of monitoring and whether appropriate patients are referred to nephrology.

**Methods:**

Clinical data from the population-based cohort Study of Health in Pomerania (SHIP-START) were matched with claims data of the Association of Statutory Health Insurance Physicians. Quality of care was evaluated according international and German recommendations.

**Results:**

Data from 1778 participants (56% female, mean age 59 years) were analysed. 10% had eGFR < 60 ml/min/1.73m^2^ (mean age 74 years), 15% had albuminuria. 21% had CKD as defined by KDIGO. 20% of these were coded and 7% self-reported having CKD. Coding increased with GFR stage (G3a 20%, G3b 61%, G4 75%, G5 100%). Serum creatinine and urinary dip stick testing were billed in the majority of all participants regardless of renal function. Testing frequency partially surpassed recommendations. Nephrology consultation was billed in few cases with stage G3b-G4.

**Conclusion:**

CKD coding increased with stage and was performed reliably in stages ≥ G4, while CKD awareness was low. Adherence to monitoring and referral criteria varied, depending on the applicability of monitoring criteria. For assessing quality of care, consent on monitoring, patient education, referral criteria and coordination of care needs to be established, accounting for patient related factors, including age and comorbidity.

**Trial registration:**

This study was prospectively registered as DRKS00009812 in the German Clinical Trials Register (DRKS).

**Supplementary Information:**

The online version contains supplementary material available at 10.1186/s12913-022-08691-y.

## Introduction

Chronic Kidney Disease (CKD) has a prevalence of approximately 10% in adults in Germany and comparable industrialised countries and is an important public health issue [1, 2]. CKD prevalence is estimated to be 27% in German general practice (GP) settings and most of these patients have early-stage CKD [3]. CKD is usually asymptomatic in the early stages and the risk of progression to end stage renal disease (ESRD) is low. Still, approximately 15,000 new patients yearly require long-term renal replacement therapy in Germany [4]. Optimal management of risk factors like hypertension and diabetes and timely referral to specialist nephrology services are deemed important to prevent progression to ERSD [2].

Although the majority of patients with CKD are cared for in ambulatory care, most research is conducted in clinical settings and there is little data on the actual management of patients with CKD in ambulatory care, regarding monitoring and management of risk factors and nephrologist referral. It is adamant to evaluate existing care, to identify processes and healthcare gaps on which to focus improvement efforts when developing and implementing healthcare policy and guidelines on CKD. Also, the collection and analysis of such data allows for the comparison of CKD management at different points in time, to evaluate the implementation of improvement measures. Clinical practice guidelines, including recommendations for the stage-appropriate management of CKD in primary care, have been developed internationally[5]. The recommendations of the Kidney Disease Improving Global Outcomes (KDIGO) guideline serve as a blueprint for such national adaptations, the British NICE guideline being a prominent example [2, 6]. In Germany, national guidelines on management of patients with non-dialysis CKD were established in 2019 for patients with CKD in ambulatory care and for the subgroup of patients with diabetes and CKD in 2010 [7, 8].

This population-based study is the first to examine real-world CKD management in the ambulatory care setting in Germany by combining claims data with clinical data including objective measures of kidney function from a cohort study in Germany. Using data from a population-based cohort with matched claims data, this study investigated how CKD is coded in ambulatory care, whether patients are aware of the diagnosis, how often and which monitoring tests are performed and whether appropriate patients are referred to nephrology. Quality of care was assessed according to recommendations from national and international clinical practice guidelines.

## Methods

### Data sources and study design

The "Study of Health in Pomerania” (SHIP) is a population-based cohort study in Northern Germany [9, 10]. It consists of three independent cohorts. For this analysis, data from the SHIP-START cohort were matched with claims data of the Association of Statutory Health Insurance Physicians using a unique random linkage number, allowing the pseudonymised claims data to be analysed together with clinical data. Declarations of consent are available for all participants as well as positive decisions from the Ethics Committee of the University of Greifswald and the regional commissioner for data protection.

**Fig. 1:**
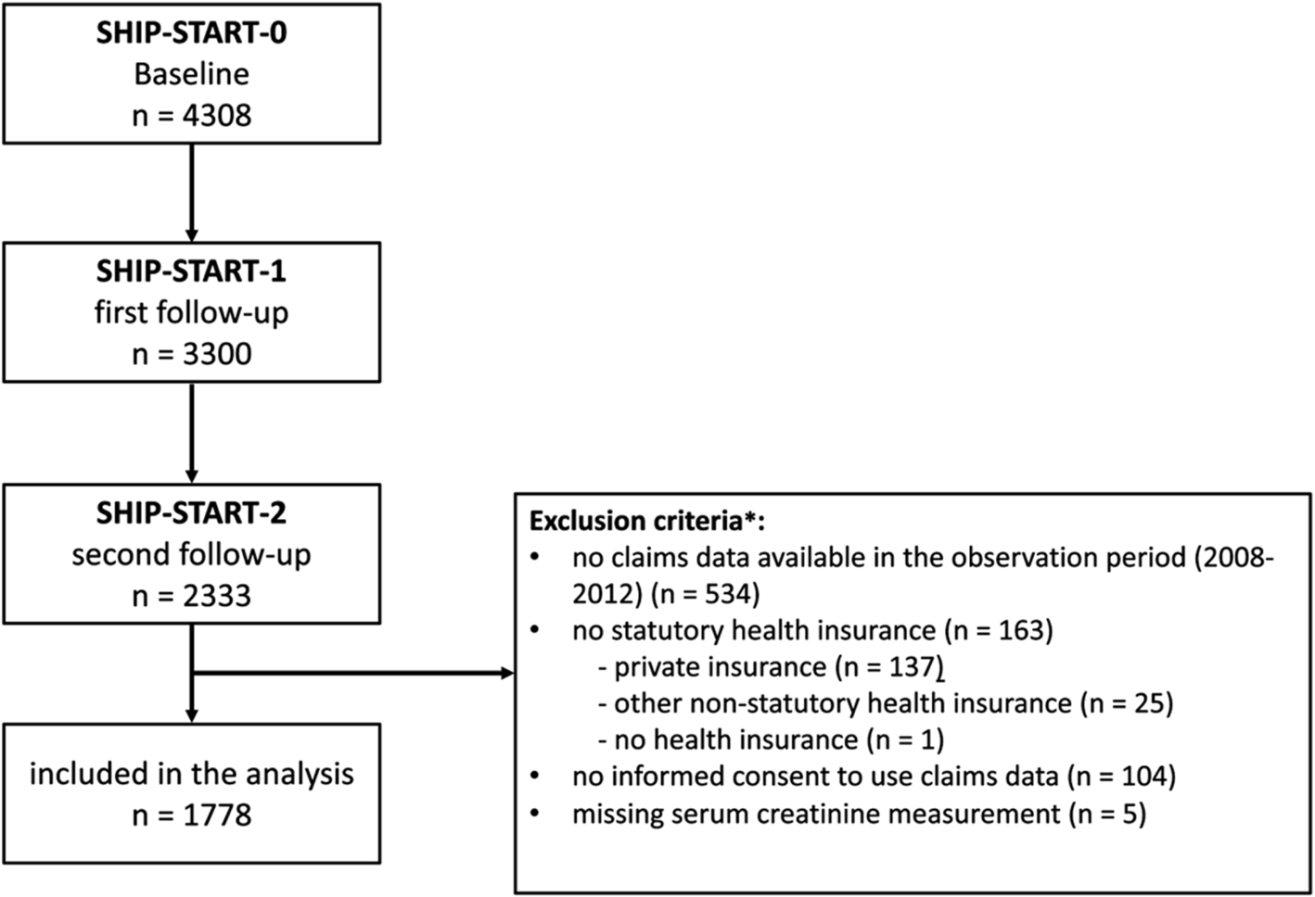
Flow chart of the study population. Data from the second follow-up of the Study of Health in Pomerania (SHIP-START-2) were analyzed. From the 2333 participants in SHIP-START-2, 1778 data sets were available for the analysis. *Of the excluded participants, some participants fulfilled more than one of the exclusion criteria

We had access to socio-demographic data, clinical parameters, laboratory data from urine and non-fasting blood samples and data from a computer-assisted personal interview from the second follow-up examination (SHIP-START-2, 2008–2012, n = 2333). Data of 1778 (76%) participants with available eGFR-values could be matched with claims data from the corresponding period. No claims data were available for participants with private or other, non-statutory health insurance, or for participants who did not consent to linkage with claims data (Fig. 1). The study relies on a single measurement of kidney function for classification of CKD, assuming that kidney functioning remains relatively constant over time. A detailed description of the SHIP study and data sources is provided in supplement 1.

#### Clinical data

The glomerular filtration rate (eGFR) was estimated using the CKD-EPI Eq [11]. The urinary albumin-creatinine ratio (ACR) was calculated for all participants with available urinary albumin and creatinine measurements in SHIP-START-2 (n = 1321). Participants were classified according to the KDIGO albuminuria categorisation [2]. Diabetes was coded when participants reported diabetes in the questionnaire, or had HbA1c ≥ 6.5%, or serum glucose ≥ 11.1 mmol/L in any of the SHIP-START surveys. Hypertension was defined as blood pressure > 140/90 mmHg, or taking antihypertensive medication. Further details concerning measurements and testing methods are provided in supplement 1.

#### Claims data

ICD-10 codes and billing codes (GOPs) were available for SHIP-START participants’ statutory health insurance during the observation period (supplement 1). Coding of CKD with ICD codes N18 and N19 (suspected or confirmed) and billing codes for laboratory tests of creatinine (32,066, 32,067), haemoglobin (32,038, 32,120), albumin (urine or serum) (32,435), calcium (32,082), parathyroid hormone (32,411), Vitamin D (32,413), phosphate (32,086) and urinary dip stick testing (32,030), urine microscopy (32,031) and microalbuminuria (32,135) were analysed. Sonographic examinations were represented by GOPs 33,042 and 33,043, nephrological co-treatment by the billing codes for nephrological care (13,591, 13,592, 13,600, 13,602, 13,610).

#### Assessment of quality of care

Assessment of quality of care is based on the recommendations of the NICE and KDIGO guidelines for the management of chronic kidney disease, including the KDIGO guideline on CKD-Mineral and Bone Disorder (CKD-MBD), as well as the German guideline on kidney disease in diabetes and the recommendations for referral of the German Society of Nephrology [2, 6, 7, 12–14].

#### Data analysis

Descriptive statistics of the study data and comparison of participants with and without available claims data were performed using R version 4.2.0 with the package kidney.epi. Correlation between eGFR and age was assessed using Spearman’s rank correlation [15, 16]. Statistical analysis for categorical variables when comparing baseline characteristics of participants with and without linked claims data consisted of Pearson's Chi-squared tests with Yates' continuity correction and effect size Cramér’s V. For continuous variables, we reported p-values derived from Mann–Whitney-U tests and the absolute value of Cliff’s delta for effect sizes.

## Results

### Sample description

**Fig. 2:**
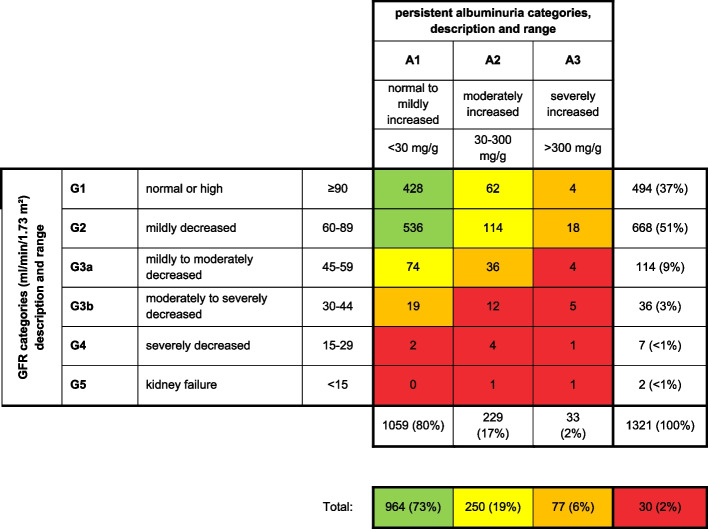
Distribution of SHIP-START-2 participants according to KDIGO prognostic categorization [2]. Categories show the adjusted relative risk relating to 5 outcomes (all-cause mortality, cardiovascular mortality, kidney failure with dialysis, acute renal failure and CKD progression) based on a meta-analysis in the general population [25]. Green: low risk, yellow: moderate risk; orange: high risk; red: very high risk. Participants in the „low risk “ group can be classified as having normal kidney function. For 457 of the 1778 SHIP-START-2 participants in this study, albumin-creatinine ratios were not available. Thus, these participants were excluded for this visualization. Percentages indicate the respective fraction of the 1321 participants with available ACR included in this figure. ACR: albumin-creatinine ratio, GFR: Glomerular Filtration Rate, SHIP-START-2: second follow-up from the START cohort of the Study of Health In Pomerania

Data from 1778 participants (age: mean 59.3 years, standard deviation ± 13.1, 56% female) were included in the analysis. 307 (17%) participants had diabetes, 1040 (58%) were hypertensive (Table 1). Figure 2 shows the classification of study participants according to KDIGO [2].

**Table 1:** Sample description

		**eGFR not impaired**	**stage** **G3a**	**stage** **G3b**	**stage** **G4**	**stage** **G5**
		*n* = 1599	*n* = 131	*n* = 38	*n* = 8	*n* = 2
**sex** female	n (%)	902 (56%)	72 (55%)	19 (50%)	3 (38%)	1 (50%)
**age**	mean (SD)	58 (12.6)	73 (6.5)	77 (7.9)	80 (8.9)	65 (14.1)
	range	31—89	56—88	57—90	66—89	55—75
**hypertension**	n (%)	894 (56%)	107 (82%)	33 (87%)	4 (50%)	2 (100%)
**diabetes**	n (%)	241 (15%)	44 (34%)	15 (39%)	6 (75%)	1 (50%)
**hypercholesterolemia**	n (%)	438 (27%)	44 (34%)	9 (24%)	3 (38%)	1 (50%)
**BMI**	mean (SD)	28.2 (4.8)	30.8 (5.3)	30.2 (4.7)	30.5 (5.8)	29.4 (13.1)
	range	16.1—49.4	20.4—44.5	20.7—42.8	23.3—38.8	20.1—38.6
**waist circumference** (cm)	mean (SD)	92 (13.9)	100 (13.6)	99 (9.9)	103 (13.3)	103 (45.9)
	range	60—145	70—140	72—127	76—114	71—136
**WHR**	mean (SD)	0.55 (0.08)	0.61 (0.08)	0.60 (0.06)	0.62 (0.07)	0.60 (0.22)
	range	0.35—0.81	0.43—0.83	0.45—0.74	0.51—0.72	0.44—0.75
**current smoker**	n (%)	326 (20%)	10 (8%)	4 (11%)	0	0

*BMI* Body mass index, (weight (kg))/(height (m))^2^, *eGFR* Estimated glomerular filtration rate, eGFR not impaired: eGFR ≥ 60 ml/min/1.73m^2^, stage G3a: eGFR 45–59 ml/min/1.73m^2^, G3b: eGFR 30–44 ml/min/1.73m^2^, G4: eGFR 15–29 ml/min/1.73m^2^, G5: eGFR < 15 ml/min/1.73m^2^, *M* Mean, *SD* Standard deviation, *WHR* Waist-to-height-ratio

### Laboratory values

Overall, 179 (10%) participants in SHIP-START-2 had a decreased eGFR (< 60 ml/min/1.73m^2^). Most participants with reduced eGFR were in stage G3a (Fig. 2) and eGFR was negatively associated with age (*r* = -0.66, *p* < 0.001). 262 participants (15%) had albuminuria and 377 (21%) were classified as having CKD because of decreased eGFR or albuminuria according to the KDIGO classification (Fig. 2).

### Self-reporting of CKD

Out of 179 participants with an eGFR < 60 ml/min/1.73m^2^, 16 (9%) reported having a CKD, of which 11 participants (69%) reported having been treated for CKD within the last 12 months. 7% (19/262) of participants with albuminuria KDIGO stage A2 or A3 reported having CKD, of which 68% (13/19) reported having been treated for CKD within the last 12 months.

### CKD coding in ambulatory care

Coding of CKD was retrieved in claims data of 5% (91/1778) of all participants during the year following the SHIP-START-2 examination, corresponding to 20% (75/377) of participants with CKD according to eGFR or albuminuria. Overall CKD was coded in 32% (57/179) of participants with eGFR < 60 ml/min/1.73m^2^, 20% (26/131) of participants in stage G3a, 61% (23/38) of participants in stage G3b, 75% (6/8) of participants in stage G4 and all participants in stage G5 (2/2) were coded (Table 2). For 18% (48/262) of participants with albuminuria, CKD was coded in the year following the SHIP-START-2 examination (Table 3).

**Table 2:** Billing and coding according to KDIGO glomerular filtration rate categorization during the year after the SHIP-START-2 examination

**number of participants coded / median number of measurements in the year after SHIP-START-2 examination**			**GFR stage (SHIP-START-2) *n* = 1778**				
			**eGFR not impaired**	**G3a**	**G3b**	**G4**	**G5**
			**(*n* = 1599)**	**(*n* = 131)**	**(*n* = 38)**	**(*n* = 8)**	**(*n* = 2)**
**ICD-10-coding of CKD** **N18.-, N19.-**		n (%)	34 (2%)	26 (20%)	23 (61%)	6 (75%)	2 (100%)
**creatinine**		n (%)	777 (49%)	98 (75%)	32 (84%)	7 (88%)	1 (50%)
		median	0	1	2	4.5	4.5
		(range)	(0 – 14)	(0 – 9)	(0 – 12)	(0 – 7)	(0 – 9)
**KDIGO guideline recommendation** (tests per year)			n.a	1–3	2–3	3–4	≥ 4
**NICE guideline recommendation** (tests per year)			n.a	1–2	2	2–3	≥ 4
**serum haemoglobin**		n (%)	672 (42%)	82 (63%)	32 (84%)	7 (88%)	0
		median	0	1	2	3.5	7.5
		(range)	(0 – 21)	(0 – 35)	(0 – 19)	(0 – 6)	(0 – 15)
**KDIGO guideline recommendation** (test per year)			n.a	1	1	2	2
**NICE guideline recommendation** (tests per year)			n.a	-	individual	individual	individual
**quantitative albumin (serum or urine)**		n (%)	28 (2%)	10 (8%)	6 (16%)	1 (13%)	1 (50%)
		median	0	0	0	0	2
		(range)	(0 – 6)	(0 – 2)	(0 – 2)	(0 – 1)	(0 – 4)
**urine testing**	**microalbuminuria dip stick testing**	n (%)	35 (2%)	13 (10%)	2 (5%)	0	0
		median	0	0	0	0	0
		(range)	(0 – 2)	(0 – 3)	(0 – 2)	-	-
	**urine dip stick testing**	n (%)	830 (52%)	88 (67%)	21 (55%)	7 (88%)	0
		median	1	1	1	3	0
		(range)	(0 – 15)	(0 – 9)	(0 – 13)	(0 – 9)	-
	**urine microscopy**	n (%)	365 (23%)	38 (29%)	13 (34%)	4 (50%)	0
		median	0	0	0	0.5	0
		(range)	(0 – 11)	(0 – 5)	(0 – 9)	(0 – 9)	-
**markers for CKD-Mineral and Bone Disorder**	**serum calcium**	n (%)	281 (18%)	42 (32%)	20 (53%)	5 (63%)	0
		median	0	0	1	1.5	0
		(range)	(0 – 9)	(0 – 7)	(0 – 12)	(0 – 6)	-
	**serum phosphate**	n (%)	44 (3%)	10 (8%)	9 (24%)	2 (25%)	1 (50%)
		median	0	0	0	0	4.5
		(range)	(0 – 3)	(0 – 4)	(0 – 7)	(0 – 2)	(0 – 9)
	**serum parathyroid hormone**	n (%)	11 (1%)	4 (3%)	4 (11%)	0	1 (50%)
		median	0	0	0	0	1.5
		(range)	(0 – 2)	(0 – 2)	(0 – 3)	-	(0 – 3)
	**Vitamin D**	n (%)	7 (< 1%)	8 (6%)	3 (8%)	0	1 (50%)
		median	0	0	0	0	0.5
		(range)	(0 – 3)	(0 – 2)	(0 – 1)	-	(0 – 1)
**abdominal or urogenital ultrasound**		n (%)	551 (34%)	63 (48%)	26 (68%)	6 (75%)	0
		median	0	0	1	1	0
		(range)	(0 – 8)	(0 – 4)	(0 – 4)	(0 – 4)	-
** ≥ 1 nephrology consultation**		n (%)	36 (2%)	3 (2%)	3 (8%)	2 (25%)	2 (100%)

ICD-coding for codes N18 (chronic kidney disease), N19 (unspecified kidney failure) and billing codes for participants according to glomerular filtration rate categorization, during the 12 months after SHIP-START-2 examination

*CKD* Chronic kidney disease, *eGFR* Estimated glomerular filtration rate, *ICD* International Classification of Diseases, *KDIGO* Kidney Disease Improving Global Outcomes, *n.a*. Not applicable, *NICE* National Institute for Health and Care Excellence, *SHIP* Study of Health In Pomerania

GFR stages; eGFR not impaired: eGFR ≥ 60 ml/min/1.73m^2^, stage G3a: eGFR 45–59 ml/min/1.73m^2^, G3b: eGFR 30–44 ml/min/1.73m^2^, G4: eGFR 15–29 ml/min/1.73m^2^, G5: eGFR < 15 ml/min/1.73m^2^

**Table 3:** Billing and coding according to KDIGO albuminuria categorization^*^ during the year after the SHIP-START-2 examination

		**albuminuria stage (SHIP-START-2)**		
**number of participants coded / median number of measurements in the year after the SHIP-START-2 examination**		**A1**	**A2**	**A3**
		**(*n* = 1059)**	**(*n* = 229)**	**(*n* = 33)**
**ICD-10-coding of CKD** N18.-, N19.-	n (%)	33 (3%)	36 (16%)	12 (36%)
**quantitative albumin (serum or urine)**	n (%)	22 (2%)	15 (7%)	4 (12%)
	median (range)	0 (0 – 6)	0 (0 – 4)	0 (0 – 2)
**microalbuminuria dip stick testing**	n (%)	31 (3%)	11 (5%)	5 (15%)
	median (range)	0 (0 – 2)	0 (0 – 3)	0 (0 – 2)
**urine microscopy**	n (%)	263 (25%)	64 (28%)	10 (30%)
	median (range)	0 (0 – 8)	0 (0 – 11)	0 (0 – 9)
**urine dip stick testing**	n (%)	565 (53%)	136 (59%)	19 (58%)
	median (range)	1 (0 – 11)	1 (0 – 13)	1 (0 – 9)
**abdominal or urogenital ultrasound**	n (%)	399 (38%)	96 (42%)	16 (49%)
	median (range)	0 (0 – 8)	0 (0 – 7)	0 (0 – 4)
** ≥ 1 nephrology consultation**	n (%)	19 (2%)	13 (6%)	5 (15%)

ICD-coding for codes N18 (chronic kidney disease), N19 (unspecified kidney failure) and billing codes for participants with available albuminuria categorization*, during the year after the SHIP-START-2 examination

Albuminuria stage A1: normal to mildly increased, < 30 mg/g, A2: moderately increased, 30–300 mg/g, A3: severely increased, > 300 mg/g; *CKD* Chronic kidney disease, *ICD* International Classification of Disease, *SHIP* Study of Health in Pomerania, *KDIGO* Kidney Disease Improving Global Outcomes

^*^Albumin creatinine ratio not available for 457 of the 1778 SHIP-START-2 participants included in this study due to missing data

### Management of CKD in ambulatory care

The proportion of patients receiving laboratory and ultrasound examinations increased with higher CKD stage (Tables 2 and 3). Serum creatinine and urinary dip stick testing were billed in the majority of all participants during the year after the SHIP-START-2 examination. Testing for microalbuminuria was performed only in the lower CKD stages. Quantitative albuminuria was rarely measured (3% of all participants).

In 3% (46/1778) of all participants and 6% (10/179) of participants with reduced eGFR, nephrological care was billed at least once during the year following the SHIP-START-2 examination (Table 2). 2% (36/1599) of participants with normal eGFR were seen by a nephrologist. In 7% (18/262) of participants with albuminuria, nephrological care was billed, as well as in 2% (19/1059) of participants without albuminuria (Table 3).

## Discussion

### Summary of the main results

Overall, 21% of the sample had CKD according to KDIGO (10% reduced GFR and 15% albuminuria). Of those, CKD was coded in 32% with reduced GFR and in 18% with albuminuria. 9% of participants with reduced GFR reported having kidney disease. Regardless of renal function, tests like serum creatinine and urinalysis were frequently billed in the majority of participants during the study period. Markers for CKD-MBD were rarely completely evaluated. For only 11% of the participants in stages G3b and G4, a nephrologist consultation was billed.

### Meaning of the results and comparison with scientific literature

CKD prevalence is generally estimated to be 10% in adults worldwide, with 3–17% in European populations [1]. In a sample of the German general population (2008–2011) of comparable age range, CKD prevalence for reduced GFR and albuminuria combined was reported to be 13%, which is significantly lower than in our analysis [17]. A comparison between SHIP-START-1 (2002–2006) and a Southern German sample (2006–2008) showed a significantly higher prevalence in Northern Germany, which may be partly explained by a different distribution of risk factors [1, 18].

#### Coding and awareness of CKD

To date, no German data were available for CKD coding and monitoring. Coding is a surrogate parameter for physicians having diagnosed the patient with CKD and incorporating this knowledge into patient management. International studies show that only 15–38% of patients with CKD are coded [19–22]. The proportion of coded CKD in our analysis was within this range (20%) and increased with diminishing kidney function. This reflects the limited clinical significance of decreased kidney function in older adults and is congruent with the findings in literature. The importance of coding for quality assessment is that when using claims data this has a large impact on the denominator for quality assessment, since only coded patients will be included. Our data suggest that claims data do not capture many patients in stage G3a.

The awareness of kidney damage was 28% in the German DEGS study (DEGS1, 2008–2011) [17]. In our analysis, only 9% with reduced GFR reported having CKD and less than half of those with stage G4 (3/8). This is in line with data from the National Health and Nutrition Examination Survey of U.S. Center of Disease Control Surveillance program for the years 2008–2012, suggesting overall CKD awareness of 10–13% in stages 3–4 with awareness increasing with stage from 3–5% in stage G3a to 38–51% in stage G4 and being negatively correlated with age[23]. The lower diagnosis awareness found in our study can be partly explained by the significantly higher age and higher rate of comorbidities of the SHIP-START population [9, 10, 24].

Our data do not allow drawing conclusions about whether the diagnosis was not made, not communicated or not remembered by the patient. From the nephrologist perspective, it is desirable for patients to be aware of their CKD [24, 25]. From a primary care perspective, other health issues are often more prominent and information about CKD does not always appear to be crucial for older people and might lead to unnecessary worries [26, 27]. On the other hand, being aware of decreased kidney function may be useful for dose-adjustment of kidney-excreted drugs and avoiding nephrotoxic substances [28]. Patients who are aware of their CKD can inform other physicians and pharmacists about their condition. However, to date, there is no evidence for a benefit of specific patient education in reducing CKD progression [29, 30]. Awareness of CKD cannot be measured with claims data.

#### Monitoring for kidney function

Serum creatinine measurements and urinary dip stick testing, which are prerequisites for staging of CKD, were performed predominantly in participants with impaired kidney function, but also very frequently in patients without renal impairment (Table 2). The frequency of measurements increased with increasing CKD stage and partially exceeded the monitoring recommendations of the KDIGO and NICE guidelines [2, 6]. These recommendations are based on expert consensus, because there are no empirical studies on monitoring intervals. The high frequency of measurements in many patients may be caused by the fact that anaemia and creatinine measurement are often ordered as part of routine laboratory testing and because creatinine used to be part of the 2-yearly preventive check-up examination (from the age of 35) and many physicians still tend to include it [31]. Additionally, it indicates a lack of coordination and communication in ambulatory care. Albuminuria was measured by urinary dip stick testing in the majority of participants. This might be due to the fact that dip stick testing is part of the preventive check-up examination. Quantitative measurement of albuminuria by ACR, as recommended by guidelines, is necessary at least once for the classification of CKD according to KDIGO [2]. The claims data indicate that quantitative albuminuria is rarely assessed. It must be kept in mind that data were collected prior to the release of the KDIGO guideline in 2012 and measurement of ACR was not formally required during the study period. Future assessment of quality of care for CKD should include at least one measurement of ACR.

#### Monitoring for CKD-MBD

The KDIGO guideline on mineral and bone disorder in chronic kidney disease (CKD-MBD) recommends with limited evidence to monitor for CKD-MBD every 6–12 months from stage G3 onwards [2, 12]. However, phosphate, calcium and parathyroid hormone assessments were significantly less frequently performed than recommended. NICE recommends monitoring only from stage G4 [6]. Awareness of monitoring for CKD-MBD seems to be low, but so is the evidence for the benefits of monitoring on clinically relevant endpoints. Therefore, measuring markers for CKD-MBD should not be part of a quality indicator set unless there is better evidence.

#### Monitoring for Anaemia

The KDIGO guideline recommends annual monitoring of haemoglobin values from CKD G3a and every six months from stage G4 [2]. NICE recommends testing patients with symptoms indicative of anaemia and recommends annual monitoring only from stage G3b onwards [6]. Actual haemoglobin testing was slightly higher than recommended by KDIGO in stage G3b and well above the recommended number of tests in stage G5. Given the high frequency of haemoglobin measurements this is a dispensable quality indicator in the German health care setting.

#### Referral to nephrology

KDIGO and NICE recommend referral to a nephrologist from stage G4, or earlier in the presence of e.g. albuminuria, persistent haematuria or progression [6, 2, 8]. The German Societies for Nephrology (DGfN) and Internal Medicine (DGIM) recommend referral from G3b, or from G3a, if additional criteria such as proteinuria or refractory hypertension are met [14]. This recommendation was not consented and is not agreed upon by German Society for General Practice and Family Medicine (DEGAM). The German guideline for Diabetes and CKD (NVL) recommends referral for persons ≤ 65 years from G3a and > 65 years from G3b [7]. International clinical practice guidelines do not distinguish between a one-time consultation to exclude specific kidney disease needing specific treatment and continuing co-treatment of patients [2, 8]. The observed nephrology consultation rate was 25% for stage G4, well below the recommendations. However, in our analysis, only 10 out of 1778 participants were in stages G4-5. A retrospective German study reports that 58% of patients with CKD in stage G1-4 had been seen by a nephrologist [32]. A similar rate was observed in an Italian study where co-treatment was reported in 56% of patients in stages G4 and above [20, 33]. From a nephrological point of view, many patients are referred too late [27]. From the primary care point of view, the progression to ESRD is a rare event and there is often no specific therapy for CKD that cannot be provided in general practice [31, 34]. The conflict in risk perception between general practitioners and nephrologists is evident when the KDIGO risk model is applied to the SHIP-START cohort (Fig. 2) [2]. This table categorises patients with warning colours in 4 risk categories and is displayed in many guidelines. Unlike one might assume this is not the risk of progressing to ESRD, but combined risk for five events (acute renal failure, CKD progression, ESRD, all-cause mortality, cardiovascular mortality) [2]. It is not quantified what is assumed to be a moderate or high risk. In fact, the risk is calculated for 1000 observed patients’ years, not adjusted for patient’s age. In this model, a patient with stage G3a and proteinuria A3 carries a risk of 5/1000 for ESRD and is classified as high risk, independent of age [2]. This is overstating the perceived and real risk of CKD and has been criticised [35].

The reasons for late or non-referral to nephrology of patients with CKD are complex as observed in our and many other studies. Causal factors that were identified were disease-specific, patient-related, as well as primary care physician related [31, 32]. Since monitoring of laboratory parameters can be carried out in general practice, patients with stable renal function and known underlying diseases may not have been referred. It is possible that some patients were seen by nephrologists before the observation period or as inpatients, which could not be recorded in our analysis. Another reason for the reluctance to refer is age-related decline of kidney function. A large proportion of people with CKD in our study were older than 75 years (Table 1). From a general practitioner's perspective, age, comorbidity, previous course and the need for a specific nephrological intervention should be incorporated for referral recommendations in addition to eGFR. Referral to nephrology co-supervision as a quality indicator for ambulatory care of patients with CKD is therefore only reasonable when a moderate performance target is set.

### Strengths and limitations

This population-based study is the first to combine claims data with clinical data including objective measures of kidney function from a cohort study in Germany. The study design allowed including patients without coded CKD according to claims data in the analysis.

Since the SHIP-START-2 examination assessed kidney function at a single time point, potential biological variation should be kept in mind when interpreting the results and especially when classifying participants into risk categories. When interpreting claims data, it should be considered that the primary purpose of coding is billing, and therefore coding quality does not allow conclusions regarding the quality of services or the consequences of monitoring tests. As a rule, monitoring and referral recommendations for CKD are largely based on expert consensus. No official consensus had been established in Germany during the study period, which limits the evaluation of quality of care. In addition, individual factors that are not included in claims data, such as patient preferences, life expectancy and social factors, may cause deviations from expert recommendations. Certain aspects of quality of care, such as blood pressure measurement, drug adaptation or therapeutic goals (HbA1c, blood pressure, cholesterol management) could not be assessed in this study based on claims data. Changes in CKD stage could not be considered in the analysis, as laboratory measurements were available for one point in time only.

With regards to albumin and creatinine measurements, the billing codes correspond to lab measurements of certain substances, but do not differentiate between measurements in serum or urine. Because measurements of creatinine in urine and albumin in serum are uncommon in the German primary care setting, we assume that creatinine measurements pertain to serum and albumin to urine samples. In this population-based sample, only eight participants had CKD stage G4 and only two participants were categorised as stage G5. This should be considered when interpreting the data.

Claims data were only available for participants with statutory health insurance who consented to data linkage. 88.1% of the German population are covered by the statutory health insurance, while 10.5% have private insurance and 1.4% are otherwise insured, e.g. in the army [36]. Private health insurance is only possible for people with higher income or for those who are self-employed. As expected, participants without claims data tended to be younger and healthier (supplement Table 1), as this group is less likely to seek healthcare. We therefore do not assume that this has a relevant impact on the generalizability of our findings.

## Conclusions

CKD coding in ambulatory care is performed more reliably in higher stages. Patient awareness of their kidney function is low and should be improved. In addition, albuminuria rarely leads to CKD coding. Assessment of renal function requires quantitative measurement of albuminuria by ACR and should be performed more frequently in patients with impaired GFR. Creatinine measurements and urinary dip stick testing are performed more often than recommended, indicating a lack of coordination of care. For measurements of quality of care regarding monitoring and referral recommendations, a consensus between general practitioners and nephrologists should be established, accounting for patient-related factors such as age.

When claims data is used as a means to monitor quality of care for patients with CKD, careful consideration of the limitations is needed. Quality of care concerning monitoring of kidney function surpassed recommendations, while anaemia and CKD-MBD could be monitored more closely. Referral seems to be performed on a pragmatic basis, and more research is needed to establish if physicians in ambulatory care sufficiently consider patient-related factors such as age, comorbidities and prognosis.

## Supplementary Information

**Additional file 1: Supplement1.** SHIP study and detailed description of data sources.

**Additional file 2: Supplemental Table 1. **Comparison of baseline characteristics of participants with and without linked claims data.

**Additional file 3: Supplemental Table 2. **Billing and coding according to KDIGO glomerular filtration rate categorization for the observation period 2008 – 2012. 

**Additional file 4: Supplemental Table 3. **Billing and coding according to KDIGO albuminuria categorization* for the observation period 2008 – 2012.

## Data Availability

Data of the SHIP study are available on request and can be applied for at https://fvcm.med.uni-greifswald.de/dd_service/data_use_intro.php. The claims data analysed during the current study are not publicly available due to legal and privacy restrictions for the protection of personal data of research participants.
