# Supplementary material for: Monitoring and management of chronic kidney disease in ambulatory care – analysis of clinical and claims data from a population-based study
Source: BMC Health Serv Res. 2022 Nov 9;22:1330. doi: 10.1186/s12913-022-08691-y (PMC9644486; doi:10.1186/s12913-022-08691-y)
Supplement: Supplementary file 1 — Additional file 1: Supplement1. SHIP study and detailed description of data sources. [file 12913_2022_8691_MOESM1_ESM.docx]

1. **Supplement 1: SHIP study and detailed description of data sources**

SHIP cohort study

In this study, we use data from the second follow-up of the first cohort (SHIP-START-2) of the Study of Health in Pomerania (SHIP), a population-based cohort study. The following descriptions were mostly derived from John et al. (2001)^1^, the SHIP data dictionary (2021)^2^, Völzke et al. (2011)^3^, Angelow et al. (2019)^4^ and Völzke et al. (2022)^5^. The SHIP study consists of independent cohorts of adults aged 20-79 years of age (at baseline) in northeastern Germany, which were drawn from the total population of West-Pomerania with stratification variables being age, sex and city/county of residence [1]. For the first cohort, SHIP-START, the study population was derived at by selecting 32 communities in the study region, from which subsequently a random sample of 7008 adults between the ages of 20-79 was extracted from the residence registries, with stratification variables being age and gender. Of the eligible persons, 126 eligible persons had to be excluded because of mortality, 615 because of migration and 1553 refused participation, resulting in a baseline cohort of 4308 participants. [1, 2]

SHIP participants are subject to repeated examinations. The baseline examinations for SHIP-START were performed between 1997-2001 (SHIP-START-0) and the follow-up examinations between 2002-2006 (SHIP-START-1) and 2008-2012 (SHIP-START-2). From the original 4308 participants, 3300 participants completed the SHIP-START-1 examination (follow-up response among still-living participants: 78.8%) and 2333 participants completed the SHIP-START-2 examination (follow-up response among still-living participants: 63.8%). [5] The following data have been collected during the study examinations:

1. Somatometric data

These data include extensive physical examinations and tests, including e.g. blood pressure, neurological examination, ultrasound, ECG, MRI, etc.

1. Interview data

Computer-assisted personal interviews of all study subjects were conducted by trained and certified interviewers. All interview data were regularly checked regarding interviewer bias during the collection phase and 10% of all interviews are sampled by independent auditors [3]. The interview data consist of numerous questions about different diseases, immunization, use of medical and dental services, medication, exercise and leisure activity, lifestyle (alcohol, tobacco), social situation and family history, sleep, workload, etc. as well as standardized tests like the minimal mental state examination.

1. Laboratory data

Non-fasting blood samples from all subjects were drawn from the cubital vein between 7 AM and 4 PM in supine position and processed according to a standardized protocol. Additionally, oropharyngeal swabs, stool and saliva samples were taken and extensive laboratory testing performed. Preanalytics and testing were performed according to standardized protocols. A detailed description was provided by Völzke et al. [3,5]

Study data

We used study data from the SHIP-START-2 follow-up.

Interview data, including sociodemographic data and data on lifetime and current smoking, were collected in the computer-assisted interview as described above. Various detailed questions on various health issues were asked and use of medication was documented. Examples of questions about CKD, hypertension and hypercholesterolemia are:

”Have you been treated medically due to this disease within the last 12 months?

… kidney disease?” [2]

“Has a raised or high blood pressure (hypertension) been determined medically since the last SHIP-study, since «MM.YYYY last examination»?” [2]

“Has a doctor prescribed anti-hypertensives in the last year?” [2]

“Have you been treated medically due to this disease within the last 12 months?

… increased level of blood lipids (cholesterol, triglycerides)?” [2]

Blood pressure was measured after a 5-minute resting phase in the right arm of seated subjects and was performed 3-fold with 3-minute intervals (HEM-705CP, Omron Corporation, Tokyo, Japan). The mean of the 2nd and 3rd blood pressure measurement was used for our analysis. Somatometric measurements were performed, which included height, weight, waist and hip circumferences. Waist circumference was measured with an inelastic measuring tape in standing patients midway between the lower rib margin and the iliac crest in the horizontal plane and rounded to the nearest 0.1 cm. Weight was measured to the nearest 0.1 kg in light clothing and without shoes. Height was measured to the nearest 1 cm using a digital ultrasound instrument. BMI was calculated as weight [kg] / (height)^2^ [m^2^].

Subjects with current tobacco use were categorized as smokers.

Serum creatinine was assessed according to the Jaffé method. Urine samples were kept at 6°C for a maximum of 2 days and albumin was assessed by BN ProSpec and Dimension VISTA (Siemens Healthcare Diagnostics, Eschborn, Germany). The lower cut-off for quantitative urinary albumin measurements was 5 mg/l albumin.

Kidney function was evaluated as estimated glomerular filtration rate (eGFR) according to the CKD-epi equation.

Claims data

Routine claims data were obtained from the regional Association of Statutory Health Insurance Physicians (KV) in Mecklenburg-West-Pomerania (MV). The KV-MV is one of 17 regional self-administrative state associations as part of the National Association of Statutory Health Insurance Physicians (NASHIP) in Germany. The regional state associations of statutory health insurance physicians are responsible for securing ambulatory healthcare, for administrative processes between physicians and the statutory health insurance, including the administration of claims data, and are involved in quality of care and revisions of the statutory fee schedules.

SHIP subjects were requested to provide written informed consent for the use of claims data for scientific research, which was provided by the great majority. 4% (104/2333) of SHIP-START-2 participants did not provide consent for linking claims data, 6% (137/2333) had private, and 1% (25/2333) had alternative non- statutory health insurance, 1 person (0.04%; 1/2333) was uninsured (**figure 1**). Data from the electronic records of the regional Association of Statutory Health Insurance Physicians Mecklenburg-West Pomerania were individually linked with SHIP data for the study period 2008-2012. Consenting participants with available claims data were identified based on a deterministic linkage using identifying characteristics (first name, surname, date of birth, place of residence, sex, insurance number, if available). For identified subjects, claims data were then matched to corresponding SHIP data using a unique, random linkage number. For 534 participants, no claims data were available from the study period.

A positive votum from the state representative for data protection and the ethics committee of the University of Greifswald was obtained in advance.

Claims data included ICD-10 diagnoses (German modification of the 10th revision of the International Classification of Diseases) and billing codes (GOP).

**Literature**

1. John U, Greiner B, Hensel E, et al. Study of Health In Pomerania (SHIP): A health examination survey in an east German region: Objectives and design. Sozial- und Präventivmedizin. 2001; 46: 186-94. 10.1007/BF01324255.
2. Study of Health in Pomerania (SHIP2) Data dictionary - Variables and their values, <https://www.fvcm.med.uni-greifswald.de/Web/DD/DD_SHIP2_englisch_10.03.2021.pdf>
3. Völzke H, Alte D, Schmidt CO, et al. Cohort Profile: The Study of Health in Pomerania. Int J Epid, 2011: 40; 294-307. <https://doi.org/10.1093/ije/dyp394>
4. Angelow A, Reber KC, Schmidt CO, Baumeister SE, Chenot JF. Prevalence of Cardiovascular Risk Factors at The Population Level: A Comparison of Ambulatory Physician-Coded Claims Data With Clinical Data From A Population-Based Study. Gesundheitswesen 2019;81:791-800. DOI: 10.1055/a-0588-4736
5. Völzke H, Schössow J, Schmidt CO, et al. Cohort Profile Update: The Study of Health in Pomerania (SHIP). International Journal of Epidemiology 2022: dyac034. https://doi.org/10.1093/ije/dyac034
