# Supplementary material for: Monitoring and management of chronic kidney disease in ambulatory care – analysis of clinical and claims data from a population-based study
Source: BMC Health Serv Res. 2022 Nov 9;22:1330. doi: 10.1186/s12913-022-08691-y (PMC9644486; doi:10.1186/s12913-022-08691-y)
Supplement: Supplementary file 2 — Additional file 2: Supplemental Table 1. Comparison of baseline characteristics of participants with and without linked claims data. [file 12913_2022_8691_MOESM2_ESM.docx]

**Supplemental Table 1: Comparison of baseline characteristics of participants with and without linked claims data**

|  |  | **claims data available** | **no claims data available** |  |  |
| --- | --- | --- | --- | --- | --- |
|  |  | n = 1781 | n = 552 | **p-value** | **effect size** |
| **sex**  female | n (%) | 999 (56%) | 236 (43%) | < 0.001 | 0.11 |
| **age** | mean (SD) | 59 (13.1) | 51 (13.6) | < 0.001 | 0.34 |
|  | range | 31 - 90 | 30 - 93 |  |  |
| **hypertension** | n (%) | 1040 (58%) | 241 (44%) | < 0.001 | 0.13 |
| **diabetes** | n (%) | 307 (17%) | 55 (10%) | < 0.001 | 0.09 |
| **hypercholesterolemia** | n (%) | 496 (28%) | 81 (15%) | < 0.001 | 0.13 |
| **BMI** | mean (SD) | 28.5 (4.9) | 27.5 (4.7) | < 0.001 | 0.12 |
|  | range | 16.1 - 49.4 | 17.3 - 46.3 |  |  |
| **waist circumference** (cm) | mean (SD) | 93 (14.0) | 91 (13.2) | < 0.001 | 0.09 |
|  | range | 57 - 145 | 62 - 133 |  |  |
| **WHR** | mean (SD) | 0.55 (0.08) | 0.53 (0.08) | < 0.001 | 0.17 |
|  | range | 0.35 - 0.83 | 0.38 - 0.80 |  |  |
| **current smoker** | n (%) | 341 (19%) | 143 (26%) | < 0.001 | 0.07 |
| **eGFR** | mean (SD) | 83.8 (18.6) | 89.4 (18.6) | < 0.001 | 0.19 |
|  | range | 9.1 – 144.9 | 5.1 – 140.2 |  |  |
| **albumin creatinine ratio** (mg/g) | mean (SD) | 45.6 (193.7) | 50.2 (363.9) | < 0.001 | 0.16 |
|  | range | 2.1 – 3768.4 | 2.2 – 6935.3 |  |  |

Comparison of demographic and clinical variables for participants with and without available linked claims data.

Statistical analysis for categorical variables consisted of Pearson's Chi-squared tests with Yates' continuity correction and effect size Cramér’s V. For continuous variables, we report p-values derived from Mann-Whitney-U tests and the absolute value of Cliff’s delta for effect sizes.

BMI: body mass index; (weight (kg))/(height (m))2, eGFR: estimated glomerular filtration rate, GOP: billing codes, ICD: International Statistical Classification of Diseases, M: mean, SD: standard deviation, SHIP: Study of Health in Pomerania, WHR: waist-to-height-ratio
