# Supplementary material for: Monitoring and management of chronic kidney disease in ambulatory care – analysis of clinical and claims data from a population-based study
Source: BMC Health Serv Res. 2022 Nov 9;22:1330. doi: 10.1186/s12913-022-08691-y (PMC9644486; doi:10.1186/s12913-022-08691-y)
Supplement: Supplementary file 3 — Additional file 3: Supplemental Table 2. Billing and coding according to KDIGO glomerular filtration rate categorization for the observation period 2008 – 2012. [file 12913_2022_8691_MOESM3_ESM.docx]

**Supplemental Table 2: Billing and coding according to KDIGO glomerular filtration rate categorization for the observation period 2008 – 2012**

| number of participants coded / median number of measurements during the observation period | |  | GFR stage (SHIP-START-2) n = 1778 | | | | |
| --- | --- | --- | --- | --- | --- | --- | --- |
|  |  |  | **eGFR not impaired** | **G3a** | **G3b** | **G4** | **G5** |
|  |  |  | **(n = 1599)** | **(n = 131)** | **(n = 38)** | **(n = 8)** | **(n = 2)** |
| ICD-10-coding of CKD  N18.-, N19.- | | n (%) | 70 (4%) | 38 (29%) | 27 (71%) | 7 (88%) | 2 (100%) |
| creatinine | | n (%) | 1409 (88%) | 129 (98%) | 37 (97%) | 8 (100%) | 2 (100%) |
|  | | median | 3 | 7 | 12 | 20 | 46.5 |
|  | | (range) | (0 - 62) | (0 - 40) | (0 - 61) | (10 - 27) | (38 - 55) |
| serum haemoglobin | | n (%) | 1370 (86%) | 125 (95%) | 36 (95%) | 8 (100%) | 2 (100%) |
|  | | median | 3 | 6 | 9 | 14.5 | 64.5 |
|  | | (range) | (0 - 58) | (0 - 182) | (0 - 63) | (6 - 25) | (61 - 68) |
| quantitative albumin (serum or urine) | | n (%) | 69 (4%) | 17 (13%) | 12 (32%) | 3 (38%) | 1 (50%) |
|  | | median | 0 | 0 | 0 | 0 | 9.5 |
|  | | (range) | (0 - 17) | (0 - 7) | (0 - 8) | (0 - 5) | (0 - 19) |
| urine testing | **microalbuminuria dip stick testing** | n (%) | 77 (5%) | 23 (18%) | 6 (16%) | 0 | 0 |
|  |  | median | 0 | 0 | 0 | 0 | 0 |
|  |  | (range) | (0 - 12) | (0 - 7) | (0 - 8) | - | - |
|  | **urine dip stick testing** | n (%) | 1271 (79%) | 114 (87%) | 31 (82%) | 8 (100%) | 1 (50%) |
|  |  | median | 4 | 6 | 8 | 13.5 | 1 |
|  |  | (range) | (0 - 82) | (0 - 31) | (0 - 34) | (4 - 32) | (0 - 2) |
|  | **urine microscopy** | n (%) | 579 (36%) | 50 (38%) | 17 (45%) | 5 (63%) | 1 (50%) |
|  |  | median | 0 | 0 | 0 | 2.5 | 0.5 |
|  |  | (range) | (0 - 46) | (0 - 25) | (0 - 24) | (0 - 21) | (0 - 1) |
| markers for CKD-Mineral and Bone Disorder | **serum calcium** | n (%) | 759 (47%) | 84 (64%) | 32 (84%) | 7 (88%) | 2 (100%) |
|  |  | median | 0 | 2 | 4 | 8 | 39 |
|  |  | (range) | (0 - 32) | (0 - 28) | (0 - 58) | (0 - 15) | (7 - 71) |
|  | **serum phosphate** | n (%) | 147 (9%) | 22 (17%) | 21 (55%) | 5 (63%) | 2 (100%) |
|  |  | median | 0 | 0 | 1 | 1.5 | 63 |
|  |  | (range) | (0 - 12) | (0 - 17) | (0 - 15) | (0 - 8) | (55 - 71) |
|  | **serum parathyroid hormone** | n (%) | 45 (3%) | 10 (8%) | 11 (29%) | 2 (25%) | 1 (50%) |
|  |  | median | 0 | 0 | 0 | 0 | 8.5 |
|  |  | (range) | (0 - 7) | (0 - 6) | (0 - 7) | (0 - 2) | (0 - 17) |
|  | **Vitamin D** | n (%) | 41 (3%) | 13 (10%) | 8 (21%) | 1 (13%) | 1 (50%) |
|  |  | median | 0 | 0 | 0 | 0 | 4 |
|  |  | (range) | (0 - 7) | (0 - 3) | (0 - 5) | (0 - 1) | (0 - 8) |
| abdominal or urogenital ultrasound | | n (%) | 950 (59%) | 98 (75%) | 36 (95%) | 8 (100%) | 2 (100%) |
|  |  | median | 1 | 3 | 4 | 5 | 1.5 |
|  |  | (range) | (0 - 32) | (0 - 20) | (0 - 18) | (2 - 14) | (1 - 2) |
| ≥1 nephrology consultation | | n (%) | 120 (8%) | 12 (9%) | 12 (32%) | 3 (38%) | 2 (100%) |

Billing codes and ICD-coding between 2008 and 2012 according to estimated glomerular filtration rate for SHIP-START-2 participants with statutory health insurance and data linkage

CKD: chronic kidney disease, eGFR: estimated glomerular filtration rate, ICD: International Classification of Diseases, KDIGO: Kidney Disease Improving Global Outcomes, n.a.: not applicable, NICE: National Institute for Health and Care Excellence, SHIP: Study of Health In Pomerania

GFR stages; eGFR not impaired: eGFR ≥60 ml/min/1.73m^2^, stage G3a: eGFR 45-59 ml/min/1.73m^2^, G3b: eGFR 30-44 ml/min/1.73m^2^, G4: eGFR 15-29 ml/min/1.73m^2^, G5: eGFR <15 ml/min/1.73m^2^
