# Supplementary material for: Monitoring and management of chronic kidney disease in ambulatory care – analysis of clinical and claims data from a population-based study
Source: BMC Health Serv Res. 2022 Nov 9;22:1330. doi: 10.1186/s12913-022-08691-y (PMC9644486; doi:10.1186/s12913-022-08691-y)
Supplement: Supplementary file 4 — Additional file 4: Supplemental Table 3. Billing and coding according to KDIGO albuminuria categorization* for the observation period 2008 – 2012. [file 12913_2022_8691_MOESM4_ESM.docx]

**Supplemental Table 3: Billing and coding according to KDIGO albuminuria categorization* for the observation period 2008 – 2012**

|  |  | **albuminuria stage (SHIP-START-2)** | | |
| --- | --- | --- | --- | --- |
| **Number of participants coded / median number of measurements during the obersvation period** |  | **A1** | **A2** | **A3** |
|  |  | **(n = 1059)** | **(n = 229)** | **(n = 33)** |
| **ICD-10-coding of CKD**  N18.-, N19.- | n (%) | 65 (6%) | 47 (21%) | 17 (52%) |
| **quantitative albumin (serum or urine)** | n (%) | 54 (5%) | 27 (12%) | 7 (21%) |
|  | median (range) | 0  (0 - 17) | 0  (0 - 19) | 0  (0 - 4) |
| **microalbuminuria dip stick testing** | n (%) | 64 (6%) | 25 (11%) | 8 (24%) |
|  | median (range) | 0  (0 - 12) | 0  (0 - 10) | 0  (0 - 8) |
| **urine microscopy** | n (%) | 396 (37%) | 89 (39%) | 18 (55%) |
|  | median (range) | 0  (0 - 46) | 0  (0 - 26) | 1  (0 - 38) |
| **urine dip stick testing** | n (%) | 834 (79%) | 191 (83%) | 29 (88%) |
|  | median (range) | 4  (0 - 70) | 5  (0 - 34) | 5  (0 - 39) |
| **abdominal or urogenital ultrasound** | n (%) | 661 (62%) | 155 (68%) | 24 (73%) |
|  | median (range) | 1  (0 - 32) | 2  (0 - 23) | 3  (0 - 14) |
| **≥1 nephrology consultation** | n (%) | 74 (7%) | 32 (14%) | 8 (24%) |

Albuminuria stage A1: normal to mildly increased, <30 mg/g, A2: moderately increased, 30-300 mg/g, A3: severely increased, >300 mg/g; CKD: chronic kidney disease; ICD: International Classification of Disease; KDIGO: Kidney Disease Improving Global Outcomes, SHIP: Study of Health in Pomerania

*Albumin creatinine ratio not available for 457 of the 1778 SHIP-START-2 participants included in this study.
